# The genome of the thin-necked bladder worm *Taenia hydatigena* reveals evolutionary strategies for helminth survival

**DOI:** 10.1038/s42003-021-02536-w

**Published:** 2021-08-24

**Authors:** Shuai Wang, Xiaolin Liu, Zhongli Liu, Yugui Wang, Aijiang Guo, Wanlong Huang, Qianhao Wang, Shaohua Zhang, Guan Zhu, Xuenong Luo, Xing-quan Zhu, Xuepeng Cai

**Affiliations:** 1 State Key Laboratory of Veterinary Etiological Biology, Key Laboratory of Veterinary Parasitology of Gansu Province, Lanzhou Veterinary Research Institute, Chinese Academy of Agricultural Sciences, Lanzhou, Gansu Province China; 2 Novogene Bioinformatics Institute, Beijing, China; 3 Key Laboratory for Zoonoses Research of the Ministry of Education, Institute of Zoonosis, and College of Veterinary Medicine, Jilin University, Changchun, China

**Keywords:** Genome, Evolutionary biology

## Abstract

*Taenia hydatigena* is a widespread gastrointestinal helminth that causes significant health problems in livestock industry. This parasite can survive in a remarkably wide range of intermediate hosts and affects the transmission dynamics of zoonotic parasites. *T. hydatigena* is therefore of particular interest to researchers interested in studying zoonotic diseases and the evolutionary strategies of parasites. Herein we report a high-quality draft genome for this tapeworm, characterized by some hallmarks (e.g., expanded genome size, wide integrations of viral-like sequences and extensive alternative splicing during development), and specialized adaptations related to its parasitic fitness (e.g., adaptive evolutions for teguments and lipid metabolism). Importantly, in contrast with the evolutionarily close trematodes, which achieve gene diversification associated with immunosuppression by gene family expansions, in *T. hydatigena* and other cestodes, this is accomplished by alternative splicing and gene loss. This indicates that these two classes have evolved different mechanisms for survival. In addition, molecular targets for diagnosis and intervention were identified to facilitate the development of control interventions. Overall, this work uncovers new strategies by which helminths evolved to interact with their hosts.

## Abstract (web summary)

Wang and colleagues report a high quality draft genome for the thin-necked bladder worm, an agricultural livestock parasitic pest. Using this draft genome and comparison with other related parasites, they reveal gene families that are selectively pressured in different species, indicative of specialised survival strategies for different hosts.

## Introduction

The thin-necked bladder worm, *Taenia hydatigena*, is a widespread gastrointestinal helminth present in livestock and wildlife. Its larval stage, *Cysticercus tenuicollis*, usually appears in the peritoneal cavity of a wide variety of animals and is easily found in meat inspections^1^. Its complete life cycle starts when intermediate hosts consume canid feces contaminated with eggs. When larvae released from these eggs penetrate the wall of the host intestine, they typically migrate to the liver and then to the mesentery/omentum, where they develop into fluid-filled bladder-like cysts. Although most infections are chronic and asymptomatic, the massive migration of cysticerci can cause acute outbreaks that result in traumatic hepatitis, severe clinical signs, and ultimately death^2^. The prevalence of *C. tenuicollis* or *T. hydatigena* is high in domestic or wild animals for vast regions, especially in developing countries. For example, the prevalence of *C. tenuicollis* in Eastern Asian countries is between 22% and 32% in pigs^1^, and *T. hydatigena* is the most common helminth species in the wolf (*Canis lupus*), with prevalence of more than 30% in different zoogeographic regions or biomes^3^. Despite this parasite being considered one of the main causes of economic and productive loss in the livestock industry^4,5^, no approved chemotherapeutic treatment is currently available against *C. tenuicollis*^2^.

The intermediate host range of *T. hydatigena* is remarkably wide, and comprises about 80 mammalian species that also include primates, as recorded in the Host–Parasite Database of the Natural History Museum of London (https://www.nhm.ac.uk/; Supplementary Data 1). Given the fact that parasites and hosts usually co-evolve and other species like *Taenia* usually have high host specificity, this variety of hosts is unusual. In accordance with its high prevalence, *T. hydatigena* is a species of great medical and ecological importance through its profound influence on diagnosis, prevalence and understanding of pathogens that cause severe human diseases^6^. First, *T. hydatigena* interferes with the serological diagnosis of cysticercosis in pigs and cattle; its frequent occurrence leads to immunodiagnostic cross-reactions with cysticercosis caused by other human *Taenia* (i.e., *Taenia solium*, *Taenia saginata*, and *Taenia asiatica*)^7,8^. Second, the infection of *T. hydatigena* can affect the prevalence and transmission of other important *Taenia* spp. pathogens through cross-protecting immunity^9,10^. Third, because of its high prevalence, its ecological niche can influence fitness and transmission dynamics of many parasites that reside in the small intestine of canids (e.g., *Echinococcus* spp. and *T. ovis*) via interspecific competition (i.e., ‘crowding effect’)^11^. Finally, due to low availability of different developmental stages for human *Taenia*, *T. hydatigena* is an ideal surrogate species to gather basic aspects of human parasite biology in cestodology, in particular of the adaptations to parasitism for these parasites.

However, the genomics of *T. hydatigena* has been little studied until now. Herein, we present a 308 Mb assembly of *T. hydatigena* genome together with transcriptomic information for several developmental stages. Analysis of the genome revealed new features for tapeworms with important implications for future research, including wide integration of viral-like elements showing similarity to sequences of vertebrate viruses, extensive alternative splicing during development, and adaptive evolutions of components with respect to tegument protection and lipid metabolism. In particular, we found that cestodes have evolved an evolutionary strategy clearly different to that in their trematode relatives, in which diversifications of an immunosuppression-related gene have been met by evolutionary gain of alternative splicing along with gene loss, rather than gene family expansions or gene retentions, which is the case for trematodes. This finding has implications for understanding how the two closely related classes fulfilled a requirement for genetic proliferation to survive in host environments and how parasites have developed sophisticated genetic systems to interact with their hosts. In addition, molecular targets for diagnosis and intervention were also identified to facilitate the development of novel interventions for cysticercosis and hydatidosis control. Overall, this work based on *T. hydatigena* genome provides insights into the evolutionary strategies for parasite survival.

## Results

### Genome assembly and gene model

Using a combination of long-read PacBio and short-read Illumina sequencing technologies, we sequenced and assembled the genome of *T. hydatigena* based on a total of 137.66 gigabases (Gb) data. The final assembly (version 1.2; unless otherwise specified, the genome assembly described in this paper refers to version 1.2) consists of 294 scaffolds (size = 308.54 Mb), with a scaffold N50 of 1.89 megabases (Mb) (Table 1 and Supplementary Data 2) and an overall %G + C content of 43.02%. More than 90% of the assembled genome is contained in 156 scaffolds. To assess the quality and completeness of this genome assembly, we back-mapped reads from transcriptomes and from small-insert libraries of genome sequencing and found that more than 94.37% and 98.69% reads were mapped, respectively, thus confirming that the assembly is highly accurate. Furthermore, CEGMA analysis indicated the presence of 89.11% core eukaryotic genes within this genome, which is comparable with other platyhelminth genomes^12–14^. BUSCO scores showed completeness (‘C’ and ‘F’) of the assembly (61.4%) comparable to the chromosome-level assembly of *Echinococcus multilocularis* (60.4%).

**Table 1:** Genomic features of *T. hydatigena* in comparison with other tapeworms.

Assembly feature	*T. hydatigena*	*T. solium*	*T. saginata*	*E. multilocularis*	*E. granulosus*	*H. microstoma*
Assembled sequences (Mb)	309	122	169	115	115	169
Repetitive content (%)^*^	63.3	13.5	33.5	13.8	13.0	24.6
Number of scaffolds	294	11,237	3626	1217	371	7
Longest scaffold size (Mb)	8.7	0.7	7.3	20.1	16.0	43.0
Scaffold N50 (Mb)	1.8	0.1	0.6	13.8	5.2	25.8
Scaffold N90 (kb)	562	5.3	29	2924	213	17,546
Contig N50 (kb)	0.3	0.1	0.5	11.3	0.2	5.8
Contig N90 (kb)	67	4	4	191	13	1209
GC content in genome (%)	43.0	42.9	43.2	42.2	41.9	36.4
Number of gene models	11,358	12,467	13,161	10,663	10,245	10,139

The statistics are based on the assembly version 1.2 for *T. hydatigena* and Wormbase ParaSite version WBPS15 for other tapeworms. ^*^The repetitive content for each genome was calculated based on the same pipeline in this study. *Mb* megabases, *kb* kilobases.

**Fig. 1:**
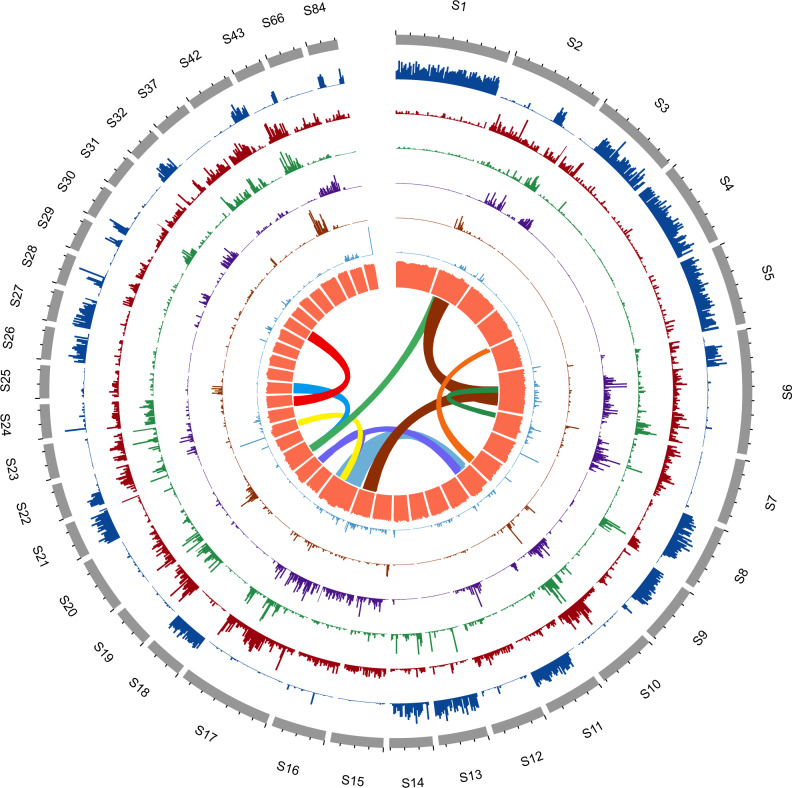
Circular overview of the *T. hydatigena* genome. Circular overview plot shows basic features across the *T. hydatigena* genome assembly. From outermost to innermost ring: density of gene models, density of long terminal repeat retrotransposons, density of DNA transposons, density of short interspersed nuclear elements, density of long interspersed nuclear elements, density of non-coding RNA, % of GC, and segmental duplications (≥5 genes in a block). All tracks except scaffold alignments show binned data with a window size of 0.1 Mb. Only scaffolds with ≥13.5 Mb in length are shown.

Predicted protein-coding and non-coding RNA genes were annotated using a combination of ab initio prediction, homology-based search, and transcript evidence gathered from RNA-seq data of multiple tissues. The overview of distribution of coding genes or non-coding genes in the genome is shown in Fig. 1. The annotation contains 11,358 non-redundant coding gene models, in line with other sequenced *Taenia* species^12,14^. A putative functional annotation was assigned to 10,325 (91%) genes based on searches against multiple functional databases, whereas 1261 genes were annotated as hypothetical proteins or proteins of unknown function (Supplementary Data 3). Furthermore, we predicted 1490 non-coding RNA genes (accounting for 0.1% of the genome), which include 34 micro-RNAs, 1223 tRNAs, 28 rRNAs, and 205 snRNAs (Fig. 1 and Supplementary Data 4). In addition, using the Hi-C technique, we scaffolded a new version of genome assembly (version 2.1). The descriptions of genome completeness, gene annotations, and repetitive elements for this genome assembly (version 2.1) are available at Supplementary Notes 1 and 2.

### Repetitive and virus-like elements

**Fig. 2:**
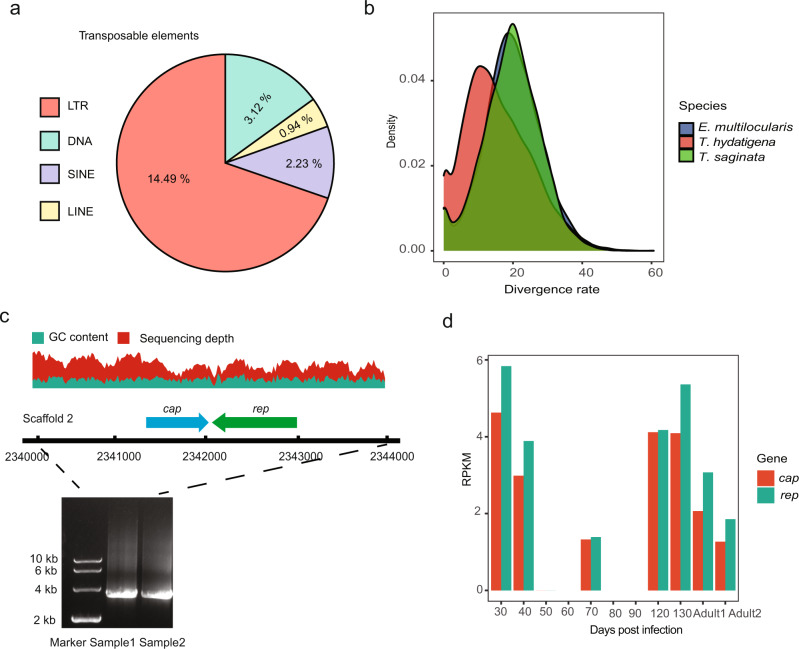
Expanded repetitive elements and virus-like sequences in the genome. **a** Schematic overview of the transposable elements repartition in the genome. Transposable elements (DNA transposons [DNA], LTR, SINE, and LINE) as a whole represent about 21% of the genome. The diagram represents the percentage of each TE category over the entire genome (%). **b** Expansion of TEs in the genome. Divergence from a consensus sequence from each element was calculated by Repeatmasker. **c** An example of *Circoviridae* virus-like sequences in the genome. GC content and sequencing depth were calculated using a window size of 20 bp. The presence of the virus genes *cap* and *rep* on the scaffold 2 was validated by genomic PCR analysis based on a primer set for amplification of the complete virus sequence and its flanking regions. Two independent samples of larvae from different hosts were used for amplifications. **d** Expression levels of the genes *rep* and *cap* of the *Circoviridae* virus sequence (in panel **c**) at different developmental stages. The *X*-axis represents the tissues of larvae collected at days post infection or adults (Adult1 and adult2 represent samples of immature proglottids and mature proglottids, respectively). FPKM values were calculated from the RNA-seq data (see ‘Methods’) of tissues.

The total assembly size is 91% of the genome size of 338 Mb estimated by a K-mer-based method (Supplementary Fig. 1). Notably, the predicted genome size of *T. hydatigena* is larger than those of *Echinococcus* (approximately 150~200 Mb) and other *Taenia* tapeworms (about 250 Mb), making it the largest genome in the family Taeniidae sequenced to date. Of the genome, 63.42% was found to be repetitive (Fig. 1 and Table 1), including representatives of many known transposable elements (TEs) (Supplementary Data 5). Approximately 14.49% of the genome is derived from long terminal repeat retrotransposons (LTRs) (Fig. 2a). The most abundant LTR retrotransposons present in the *T. hydatigena* genome are *Gypsy* elements (6.58% of the genome), followed by *Copia* (1.37%) and *ERV1* (0.40%). Consistent with previous studies that ancient expansion events occurred in *Taenia* species^13^, our analysis also identified a peak of divergence rate of TEs for the *T. hydatigena* genome (~0.2 Kimura substitution rate) (Fig. 2b). However, the *T. hydatigena* genome suggests lineage-specific sequence evolution in this species, as it contains more recent expansions of TEs than other Taeniidae genomes (e.g., *T. saginata* and *E. multilocularis*). In addition, local segmental duplications (≥5 genes in a genomic block) were found in 0.63% of the genome (Fig. 1). Most of the repeat elements, which account for 46.68% of the genome, are not homologous to any known repeat element classes (‘Class = Unknown’) (Supplementary Data 5) and these unclassified repetitive contents contribute to most of the overall expanded genome size of *T. hydatigena*. Some of these repeat content have been highly expanded in the *T. hydatigena* genome, such as ‘rnd-5_family-1246’ (0.37% of the genome), ‘rnd-6_family-990’ (0.31%), and ‘rnd-5_family-857’ (0.29%) (Supplementary Data 6).

Intriguingly, analysis of interspersed repeat elements revealed a widespread presence of endogenized viral sequences in the *T. hydatigena* genome. Genome-wide profiling identified integrations of 38 virus-like sequences (VLSs) that have been potentially transferred horizontally from viruses (Supplementary Data 7). The most widespread VLSs were closely related to invertebrate-infecting DNA viruses, such as decapod penstyldensovirus, dragonfly cyclovirus, and white spot syndrome virus. However, others show similarity to sequences of vertebrate viruses, such as feline cyclovirus, camelpox virus, bat circovirus, and simian immunodeficiency virus. It is unlikely that the presence of these VLSs in the genome is due to contamination or active viral infections in the materials for sequencing, since almost uniform sequencing coverages along the bodies of these VLSs and their flanking regions were detected. Also, these VLSs were typically truncated or fragmented, suggesting an ancient integration origin. Interestingly, some members of these endogenous VLSs (39.72%) were transcriptionally active, as shown by RNA-seq data (genomic loci were 100% covered by the RNA-seq reads and RPKM value ≥ 10 in at least one tissue) (Supplementary Data 8). For example, several feline cyclovirus replication (*rep*) gene-related sequences were measurably expressed. These sequences are usually located within a putative complete viral genome structure consisting of two major open reading frames (ORFs) (*rep* and *cap*) (Fig. 2c, d), which are arranged in opposite directions and typical for *Circoviridae* viruses. We speculate that these sequences are derived from past viral infections of tapeworms.

### Extensive alternative splicing events during larval migration

**Fig. 3:**
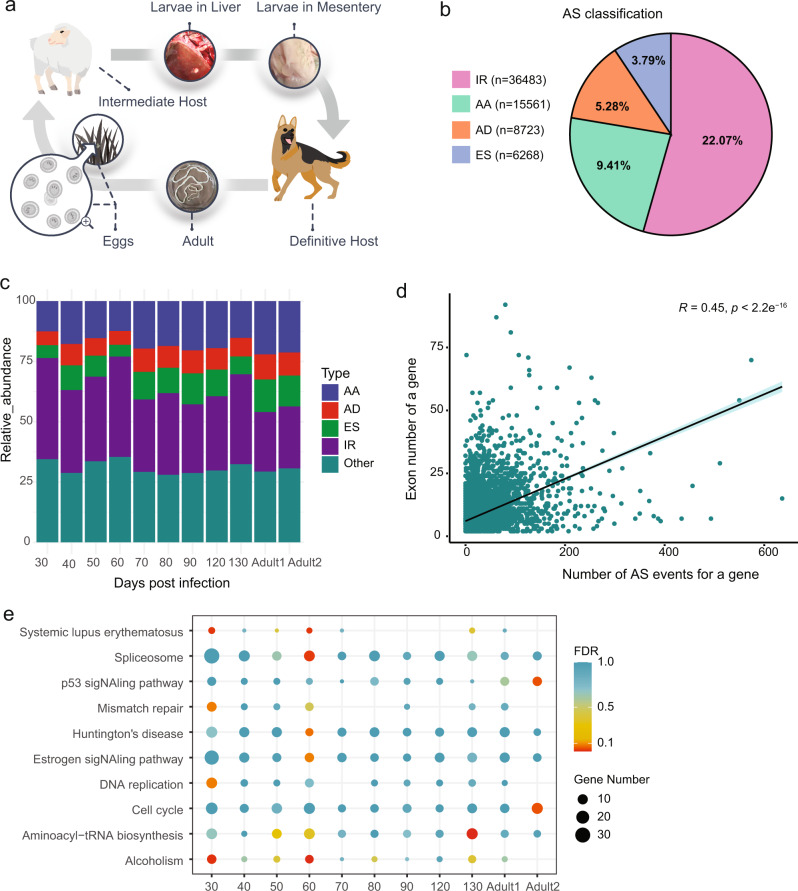
Extensive alternative splicing events in transcriptomes. **a** The typical life cycle for *T. hydatigena*. Its complete life cycle starts from eggs that are consumed by intermediate host (e.g., sheep). Larvae penetrate the wall of host intestine, and typically migrate to the liver and then mesentery/omentum, where they develop into fluid-filled bladder-like cysts. The adult tapeworms in canids (e.g., dogs) produce proglottids that are then passed in the feces. **b** Alternative splicing event types identified in the genome. The diagram represents the percentage of each alternative splicing category (intron retention [IR], alternative acceptor sites [AA], alternative donor sites [AD], and exon skipping [ES]). **c** Relative abundances of alternative splicing types for each sample of different developmental stages. The *X*-axis represents the tissues of larvae collected at days post infection or adults (Adult1 and adult2 represents samples of immature proglottids and mature proglottids, respectively). **d** Correlation between alternative splicing event number and exon number in the genome. *R* and *p* denote Pearson’s correlation coefficient (two-sided, confidence level = 0.95) and the corresponding *p*-value, respectively. **e** Enriched pathways of KEGG for the genes with abundant alternative splicing events. The genes that have more than 5 alternative splicing events detected in all the samples in RNA-seq analysis were used for the enrichment analysis. FDR was used to correct the multiple hypothesis testing.

RNA-seq was used to analyze gene expression in tissues of multiple parasite life-stages, involving proglottids of the adult parasite and cysts on the surface of the liver and the abdominal cavity of the host (sheep) (Fig. 3a). We found significant expression for 10,654 genes (FPKM ≥ 1) in total from all the tissues studied, with between 7890 (adult neck tissues) and 9571 (larval tissue of 60 days post infection) genes expressed at each stage. Alternative splicing is a major process underlying the generation of proteomic complexity. For these expressed genes, we identified 16,528 alternative splicing events from the transcripts obtained from 11 tissues or stages (mature or immature proglottids of adults and larvae at days 30, 40, 50, 60, 70, 80, 90, 120, and 130 post infection in sheep), involving 7049 multi-exon genes (68.27% of total genes; 76.98% of the multi-exon genes). Overall, the predominant alternative splicing type in *T. hydatigena* is intron retention (22.07%), followed by alternative acceptor site (9.41%), alternative donor site (5.28%), and exon skipping (3.79%), respectively (Fig. 3b).

We observed a marked variation of alternative splicing-type abundances across developmental stages (Fig. 3c). Alternative acceptor site is more prevalent in adult tissues, whereas intron retention is the most common type in larvae. The latter changed during the development stages of the larvae between days 30 and 40, roughly corresponding to the migration from the digestive tract to liver, and between days 50 and 60, when the parasite settles in the abdomen. This correlation suggests a link of this dominant alternative splicing type with larval development and invasion. Alternative splicing event numbers seem to positively correlate with intron length (*r* = 0.19, *p* < 2.2e^−16^), and exon number (*r* = 0.45, *p* < 1e^−16^), but negatively correlate with GC content (*r* = −0.098, *p* < 2.2e^−16^) (Fig. 3d). Genes with abundant alternatively spliced transcripts (≥5 alternative splicing events per gene) were overrepresented for functions in the cell cycle, including DNA replication, mismatch repair, and p53 signaling pathway (Fig. 3e).

### Alternative splicing and gene loss associated with tapeworm survival

**Fig. 4:**
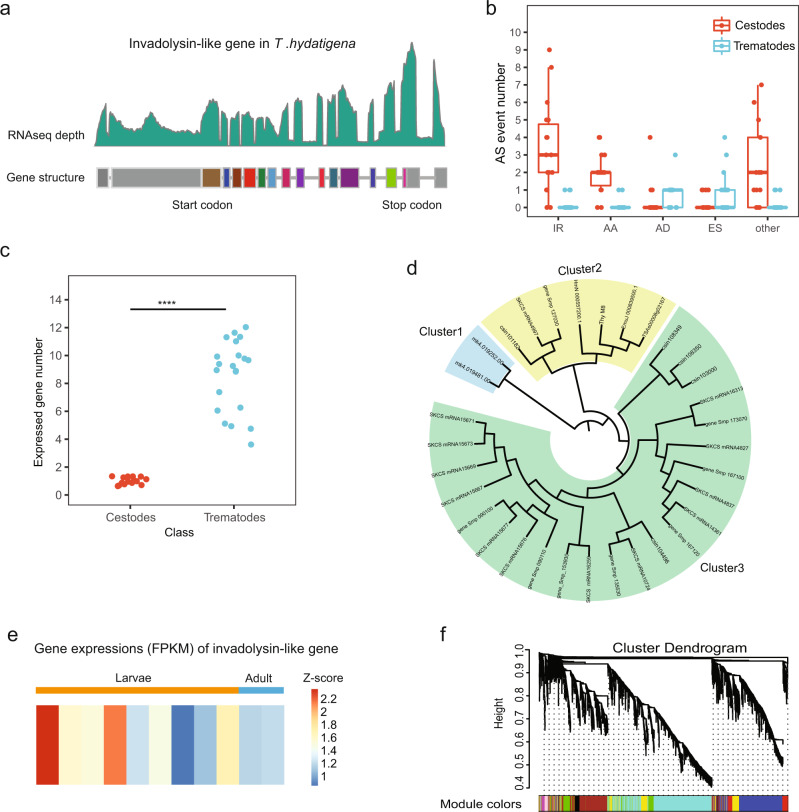
Invadolysin-like genes in *T. hydatigena* and other flatworms. **a** The gene structure of the gene encoding ‘invadolysin’ (M08 peptidase) in *T. hydatigena*. The gene structure was manually curated based on RNA-seq data and gene model prediction. Splice junctions were manually confirmed using the alterative splicing information identified from the RNA-seq data. The gray regions indicate untranslated regions (UTRs). **b** Alternative splicing events detected in samples of cestodes (*T. hydatigena*, *T. saginata*, *T. asiatica*, and *T. solium*) are significantly higher than the samples of trematodes (*S. mansoni*, *S. japonicum,* and *C. sinensis*). Available samples at different life stages for each species were used for alternative splicing event identification (see ‘Methods’). Few alternative splicing isoforms of M08 peptidases were detected from trematodes. **c** The expressed (sequencing depth >1) gene members of M08 peptidase family at different developmental stages for each involved species in the cestodes (*n* = 1 for *T. hydatigena*, *T. saginata*, *T. asiatica*, and *T. solium*) are less than those in the trematodes (*S. mansoni, S. japonicum*, and *C. sinensis*). **d** Phylogenetic tree for the genes encoding ‘invadolysin’ in platyhelminths. Cluster 1 consists of invadolysin-like genes identified in the genome of *S. mediterranea*; cluster 2 consists of invadolysin-like genes identified from the genomes of cestodes and trematodes; cluster 3 only consists of invadolysin-like genes identified from the trematode genomes. **e** Expression levels (FPKM) of the invadolysin-like gene in *T. hydatigena*. **f** The invadolysin-like gene in *T. hydatigena* was clustered into a module (turquoise) in WGCNA analysis. This module showed a higher eigengene expression level at days 30 and 60 post infection in the intermediate host (sheep). The whiskers of boxplot indicate 1.5 × interquartile range. ^****^p < 0.0001 using the two-sided Wilcoxon rank sum test (confidence level = 0.95).

Included in the top genes with abundant splice isoforms is the gene encoding invadolysin (M08 peptidase), which has an N-terminal signal sequence, may be a surface or secreted protein, and may facilitate invasion of parasites through its immunosuppressive properties^15,16^. In schistosome genomes, this gene’s family has expanded into multiple copies (e.g., more than 10 in *Schistosoma mansoni*^17^) that differ in their exon–intron structure and basal expression. In contrast, only one invadolysin gene was identified in the genomes of tapeworms sequenced to date (Supplementary Data 9). After manual validations of the gene structure, we found this gene in the *T. hydatigena* genome contains 14 exons with a putative coding sequence between exons 2 and 14 (Fig. 4a). According to the alternative splicing event analysis of RNA-seq data, this gene produces a large group of isoforms (at least *n* = 58) from a single locus in the genome, with intron retention as the most abundant alternative splicing type. This is consistent with the hypothesis that these tapeworms fulfil the requirement of functional proliferation for this gene by use of alternative splicing, in contrast to the use of multiple gene copies in Schistosomes. In accordance with this hypothesis, alternative splicing events were found to be rare for the M08 family in trematodes (Fig. 4b), whereas more family members were expressed in each stage of trematodes (Fig. 4c).

We next sought to infer the evolution of this gene in the genomes of the two evolutionarily closely related parasite classes. The phylogenetic analysis by comparing the gene tree (Fig. 4d) and species tree supported gene loss events along the ancestral cestode lineages and clearly indicated that the gene duplications are specific for trematodes. Thus, it is likely that the gene loss event occurred after the split between Cestoda and Trematoda but before the diversification of the cestode species (loss of the blue cluster), which supports that the two parasite classes employed different evolutionary strategies.

The invadolysin-like gene was expressed in abundance throughout all the 15 tissues and showed a higher expression level in larval stages (average FPKM = 50.75) than in the adult stage (FPKM = 16.33) (Fig. 4e, heatmap). This suggests that this invadolysin-like gene is more active during the larval invasion process. Additionally, our WGCNA results revealed that this gene is clustered into a module (turquoise) that showed a higher eigengene expression level at days 30 and 60 post infection in the intermediate host (sheep) (Fig. 4f, Supplementary Fig. 2, and Supplementary Data 10), i.e., during the larval migration to liver or abdomen. GO enrichment analysis revealed the genes in this module are enriched in biological processes associated with ‘lipid metabolism’, such as ‘phospholipid metabolic process’, ‘cellular lipid metabolic process’, and ‘glycerophospholipid metabolic process’ (false discovery rate, FDR < 0.1) (Supplementary Data 11). Together with the expression pattern, this evidence suggests that this module is involved in the migration to these lipid-rich organs and that the ‘invadolysin’-like gene is important for larval survival during migration to these new organs. Collectively, our results suggest that trematodes and cestodes might have developed distinct genetic programs to circumvent the barriers imposed by the hosts’ immune system.

### Genome adaptations in surface components and lipid metabolism

**Fig. 5:**
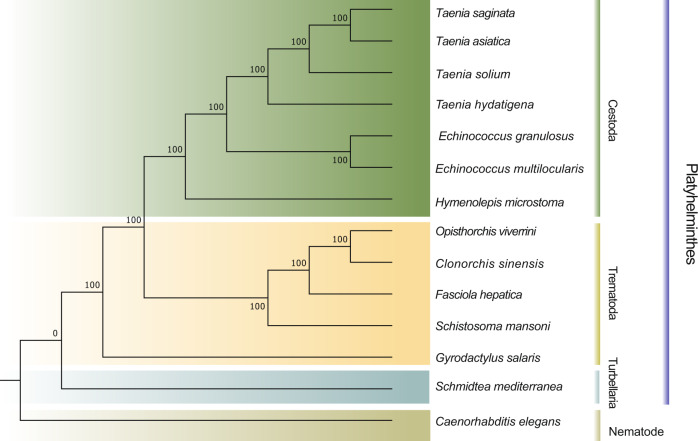
Phylogenetic relationship between *T. hydatigena* and other worms. The phylogeny was inferred from concatenated proteins of single-copy ortholog genes (*n* = 253). The branch lengths of the tree are not proportional to substitutions per site. The gene sets used in this analysis were retrieved from Wormbase (WBPS14).

The wide host range of *T. hydatigena* is indicative of a very special ecological niche. To better understand the evolution of the *T. hydatigena* genome through the analysis of major evolutionary events, we performed a comparative analysis with 14 other helminth genomes and identified 253 one-to-one orthologous genes in these 14 species. Phylogenetic analysis using a maximum likelihood method confirmed the taxonomic affiliations with other *Taenia* tapeworm species (*T. solium/saginata/asiatica*) (Fig. 5). Comparative analysis revealed that 107 and 16 gene families along the *T. hydatigena* lineage underwent either significant expansion or contraction, respectively, after divergence from other *Taenia* species (Supplementary Data 12). The highly duplicated, expanded families included genes encoding the following products: tapeworm-specific surface antigens (e.g., oncosphere proteins TSOL16B and Tsol15); heat shock cognate protein (HSP70 and HSP90); tegmental components (such as β−1,3-galactosyl-*O*-glycosyl glycoprotein, collagen alpha-3(VI) chain); and glycosylases likely to have tegmental components as substrates (such as glycosyl-transferase 14 family member) were highly duplicated. One of the most striking gene family expansions in the tapeworm genome was a family of high-cysteine chorion (eggshell) protein (*n* = 40), well known for protective roles in silkworms.

In comparison with other *Taenia* relatives, *T. hydatigena* cysticercus has preference for omentum and mesentery, both rich in fat and lipids. Consistent with this phenomenon, we observed striking adaptive evolution in genes related to lipid absorption and metabolism. The *T. hydatigena* genome has greatly expanded gene families encoding fatty acid binding proteins (*n* = 16) and phosphatidate phosphatases (*n* = 5), suggesting an increased ability to ingest and metabolize lipids. We detected strong positive selection signals enriched in the key genes with regard to lipid transport (lipid transport protein N-terminal), fatty acid elongation (long-chain-fatty-acid–CoA ligase), lipid metabolism (phosphatidate phosphatase), propanoate metabolism (L-lactate dehydrogenase B chain and acetyl coenzyme A synthetase cytoplasmic), lipolysis in adipocytes (insulin growth factor 1 receptor beta, phosphatidylinositol-4,5-bisphosphate 3-kinase, and receptor-type guanylyl), phosphatidylinositol signaling system, and other lipid metabolism-related processes (Supplementary Data 13). Most of the above are key regulators of lipid homeostasis and central to energy generation processes. Interestingly, about 70% of these genes have undergone ≥5 alternative splicing events per gene in the developmental stages, further indicative of their critical roles in the lifecycle of *T. hydatigena*. In particular, this was observed in genes involved in lipid metabolism, such as those coding for lipid transport protein N-terminal, and long-chain-fatty-acid–CoA ligase. These rapidly evolving genes may contribute to adaptation to the specialized environment.

### Molecular targets for diagnosis and intervention

*T. hydatigena* shares intermediate hosts with *T. solium* (pigs) and *Echinococcus granulosus* (sheep), which are of great medical and economic importance and need to be eradicated. The overlap of ecological niches between larval cestodes of these parasites gives rise to an issue of cross-reactions occurring during serodiagnosis for animals with infections. In order to provide antigen candidates that can be used in a specific, serological, diagnostic test, we searched species-specific genes potentially valuable in developing molecular and/or immunological diagnostic tools, particularly those for specific detection of *T. hydatigena*. The analysis yielded 10,760 (85%) proteins in *T. hydatigena* that are homologous to proteins in *T. solium* and *E. granulosus* (E-value ≤ 1e^−5^, identity ≥ 50%) (Supplementary Fig. 3). Searching for species-specific protein-coding genes in *T. hydatigena*, we observed 668 such genes by comparison with *T. solium* (Supplementary Data 14) and 1579 genes by comparison with *E. granulosus* (Supplementary Data 15). By intersecting these two sets of species-specific genes, we identified 598 genes specific in the *T. hydatigena* genome by comparison with both of these tapeworms (Supplementary Data 16). These genes are potential candidates for use in differential diagnosis. However, the feasibility of these genes in developing immunological approaches needs experimental evaluation. We also identified molecular targets for intervention. From the *T. hydatigena* genome, 73 G-protein-coupled receptors (GPCRs), 317 protein kinases, and 59 ligand-gated ion channels (LGICs), which are well-known drug targets and may constitute molecular targets for intervention, were identified based on homology searches (Supplementary Data 17–19). Most of these drug targets are conserved in tapeworm genomes and represent key participants in terms of the invasion, migratory, and digestive processes of *T. hydatigena*.

## Discussion

We report the first whole-genome sequence of the thin-necked bladder worm, *T. hydatigena*, a major causative agent for cysticercosis in livestock worldwide. Our data recapitulate some well-characterized genome features in tapeworms^12–14^ and also offer insights into the mechanisms underlying parasite adaptations. This is facilitated by revealing widespread presence of endogenized *Circoviridae* sequences, the extensive alternative splicing events associated with migration in host tissues, and the adaptation in genes of tegmental components and lipid metabolism. In particular, the analysis of alternative splicing events along developmental stages highlighted a novel evolutionary strategy for the invadolysin-like gene potentially involved in immune suppression in tapeworms, which is distinct from that in trematodes.

The *T. hydatigena* genome reveals remarkable features of tapeworm genomes. *T. hydatigena* has the largest genome size within the *Taenia* species that has been sequenced to date. Although it is unclear how this happened, our results revealed that it is at least in part attributable to expansions of retrotransposable elements, rather than large-scale segmental duplication events. Given that fitness‐based selection and phenotypic plasticity are connected to genome size^18–20^, this expanded genome may be related to its strikingly wide host range. Notably, the wide endogenization of various viral genomes into the tapeworm genome suggests previous viral infections^21^, consistent with a recent study that identified six novel RNA viruses that infect the tapeworm *Schistocephalus solidus*^22^. Importantly, the study proved that the *S. solidus* rhabdovirus can be transmitted from tapeworms to their parasitized hosts, impacting *S. solidus*–host interactions. In this study, we have identified multiple VLSs that are closely related to vertebrate viruses, strongly suggesting that parasites potentially serve as a virus reservoir that may have influence on their hosts and host–parasite interactions. It remains unclear how these VLSs were endogenized into the *T. hydatigena* genome. Several studies have reported horizontal transfers of TEs between parasites and their hosts^23–25^. For instance, RTE1_Sar was probably horizontally transferred between parasitic nematodes and the common shrew *Sorex araneus*^23^. Parasites have long lasting physical contact with their hosts, increasing the chances of horizontal transfer of TEs or virus elements. Therefore, it is possible that these vertebrate virus-related VLSs were horizontally transferred from a vertebrate host into the genome of *T. hydatigena*. The fact that putative complete viral genome structures are present suggests that some of the VLSs are derived from integrations of entire viruses instead of partial sequences.

The evolution that occurred after the split from other cestodes indicates that *T. hydatigena* has undergone some adaptive evolutions to its specialized niches (e.g., preference of living in omentum and mesentery). Our results reveal that genes encoding surface and lipid metabolism involving proteins are closely related to these processes. This is in accordance with the previous findings for tapeworms that these components probably have influence on tapeworm fitness^12^. For example, in *T. hydatigena* we found strong adaptative signals in the components of tegument or surface (e.g., tapeworm-specific surface antigens, collagen alpha-3[VI] chain, and glycosyltransferase 14 family member) or in the lipid uptake/metabolism (e.g., proteins related to fatty acid binding, and fatty acid elongation), which have been linked to adaption to new environments. Our findings assign key roles for survival to these components in tapeworms.

Alternative splicing is a major mechanism to generate genetic material^26^. Here, we found this mechanism expands the coding capacity of the parasite genome and is associated with parasite invasion. The types of alternative splicing were dependent on the stages when the parasite larvae migrate to different tissues or organs, which involves genes related to ‘cell cycle’. This suggests that alternative splicing facilitates survival in different environments. More importantly, analysis of alternative splicing events for the invadolysin-like gene with respect to immunosuppression highlights that extensive alternative isoforms of a singleton are also linked to the survival of cestodes. It is of interest that the close relatives of cestodes, trematodes, fulfil the demand of functional proliferation for this gene by duplication, whereas only a few alternative splicing events occur. Since alternative splicing and gene duplication are inversely correlated evolutionary mechanisms^26^, our findings support a notion that selection for genic proliferation in the parasitic flatworms might be interchangeably met by either evolutionary mechanism. However, it is still unclear how gene duplicates lost splice variants in trematodes and how the singleton acquired splice variants in cestodes. Phylogenetic analysis of the M08 peptidase family suggests that gene family contraction occurred in the cestode lineage, which might make the demand of function diversifications rely mainly on the singleton. This implies that splice-variant acquisition, rather than loss, is a better explanation for our results. This is a valuable example for the understanding of evolutionary divergence between cestodes and trematodes.

In summary, this report highlights novel details on the biological properties of helminths, reveals insights on evolutionary strategies for helminth survival, and would serve as a unique resource to the scientific community for future experimental research of molecular mechanisms.

## Methods

### Sample collection and sequencing

An adult worm of *T. hydatigena* was collected from a naturally infected dog (Lanzhou, Gansu Province). Larvae (cysticerci) of *T. hydatigena* were obtained from sheep experimentally infected with the eggs (*n* = 9 × 10^4^) in proglottids of the adult worm. The animals were cared in accordance with good animal practice according to the Animal Ethics Procedures and Guidelines of the People’s Republic of China. The study was approved by the Institutional Committee for the Care and Use of Experimental Animals of Lanzhou Veterinary Research Institute, Chinese Academy of Agricultural Sciences (no. LVRIAEC2010-002). Genomic DNA was extracted from neck and middle proglottids (immature) using the DNeasy Blood & Tissue Kit (Qiagen, China) according to the manufacturer’s instructions, for constructing multiple sequencing libraries, respectively. Specifically, seven paired-end Illumina sequence libraries with different length insert size (250 bp, 350 bp, 450 bp, 2kb, 5 kb, 10kb, and 15 kb) and one PacBio library was constructed using the extracted DNA, which were sequenced on Illumina HiSeq X platform and the PacBio Sequel platform, respectively. After sequencing, the raw sequencing data generated by Illumina HiSeq X platform were filtered for adapters, reads with N bases of more than 10%, and reads with low-quality bases (≤5) of more than 50%, and PacBio data were filtered with the default parameters of the platform, which resulted in 128 Gb high-quality paired-end Illumina reads (a coverage of ~ 378×) and 11 Gb high-quality Pacbio reads (a coverage of ~33×), respectively.

For RNA-seq, samples of *T. hydatigena* were collected at different developmental stages from sheep abdominal cavity, including adults (one mature and one immature proglottids) and larval stages. The latter consisted of samples on the surface of liver at days 30 and 40 (one each) post infection in sheep, and on mesentery/omentum in the abdomen at days 50, 60, 70, 80, 90, 120, and 130 post infection in sheep (*n* = 2, 1, 2, 1, 3, 1, 1, respectively). Total RNA was extracted using TRIzol reagent (OMEGA, USA) following the manufacturer’s recommendations. Sequencing libraries were generated using NEBNext^®^ UltraTM RNA Library Prep Kit for Illumina^®^ (NEB, USA) following manufacturer’s recommendations. All these libraries for RNA-seq were sequenced on Illumina HiSeq platform with PE150.

### Genome assembly

The genome size of *T. hydatigena* was estimated using a K-mer-based method^27^. Distribution of minor allele frequency of the *T. hydatigena* isolate was estimated (Supplementary Fig. 4). The genome was assembled using a combination of long-read PacBio and short-read Illumina sequencing technologies. First, overlap-layout-consensus approach implemented in Canu (v1.3)^28^ is used for assembling the PacBio long reads. Then, the short reads from Illumina were used to correct any remaining errors using Pilon (v1.22)^29^. Finally, contigs were assembled into scaffolds using Mate-Pair reads (with library insert sizes of 2 kb, 5 kb, 10 kb, and 15 kb) by SSPACE (v3.0)^30^. These processes yield a final *T. hydatigena* genome assembly (version 1.2), which is used for the comparative analyses in this study. In addition, using the Hi-C technique, a new version of genome assembly (v2.1) is now available (see Methods and statistics in Supplementary Note 1).

In order to assess nucleotide accuracy of the genome assembly, Illumina short reads were mapped to the *T. hydatigena* genome assembly using BWA (v0.7.8)^31^ with default parameters and performed variant calling with Samtools (v0.1.19)^32^. Completeness of genome assembly was assessed by the following methods: First, Core Eukaryotic Genes Mapping Approach (CEGMA)^33^ (*n* = 248 CEGs) and Benchmarking Universal Single-Copy Orthologs (BUSCO)^34^ (*n* = 954 BUSCOs, metazoan_odb10) were used to assess the completeness of the genome. Second, short reads from a library with a small insert size (350 bp) and RNA-seq reads were mapped back to the scaffolds using BWA (v0.7.8), respectively.

### Genome annotation

Repetitive sequences within the *T. hydatigena* genome and the genomes of other tapeworms (*T. saginata, T. solium, E. multilocularis, E. granulosus,* and *H. microstoma*) were predicted by both homologous-based and ab initio-based methods. For homologous searches, RepeatMasker (v4.0.5) (http://repeatmasker.org) and RepeatProteinMask^35^ were used for aligning the genome sequences against Repbase database^36^. For ab initio prediction, LTR_FINDER (v1.07)^37^, RepeatScout (v1.0.5)^38^, and RepeatModeler (v1.0.4) (http://repeatmasker.org/RepeatModeler/) were firstly used for predicting repetitive elements to construct a custom library for Repeatmasker. In addition, the tandem repeats were predicted using Tandem Repeats Finder (v4.07b)^39^. MCScanX^40^ was used to detect blocks (≥5 genes in a block) of segmental duplications.

The gene models of coding genes in the genome were predicted by Evidence Modeler (v1.1.1)^41^, integrating evidence from ab initio predictions, homology-based searches, and RNA-seq alignments. For ab initio annotation, Augustus (v3.1)^42^, GeneID (v4.1)^43^, GeneScan^44^, GlimmerHMM (3.0.4)^45^, and SNAP (2013-02-16)^46^ were simultaneously used to predict coding regions in the repeat-masked genome. Of these, Augustus, SNAP, and GlimmerHMM were trained by PASA-H-set gene models (PASA Trinity set). For homology-based prediction, protein repertoires of *T. asiatica* (PRJNA299871), *T. saginata* (PRJNA71493), *T. solium* (PRJNA170813), *E. multilocularis* (PRJEB122), *S. mansoni* (PRJEA36577), *H. microstoma* (PRJEB124), *M. corti* (PRJEB510), *S. japonicum* (PRJEA34885), *S. haematobium* (PRJNA78265), *C. briggsae* (PRJNA20855), and *C. elegans* (PRJNA13758) were aligned against the *T. hydatigena* genome using TBLASTN. GeneWise (v2.4.1)^47^ was used to predict the exact gene structure of the corresponding genomic region for each BLAST hit. For transcriptome-based prediction, RNA-seq reads were directly mapped to the genome using HISAT2 (v 2.0.4) and the mapped reads were assembled into gene models by Cufflinks (v2.1.1). Evidence Modeler (v1.1.1) was used for integrating all the evidence for each gene model. For non-coding genes, the gene structures of transfer RNAs (tRNA) were identified using tRNAscan-SE software (v1.3.1)^48^. The rRNA fragments were predicted by searching against the invertebrate rRNA database using BLAST (E-value ≤ 1e^−10^). The microRNA (miRNA) and small nuclear RNA (snRNA) genes were predicted by INFERNAL (v1.1rc4)^49^ using Rfam database^50^. The predicted protein-coding genes in the *T. hydatigena* genome were functionally annotated based on homologous searches against databases of SwissProt, NCBI ‘nr’ database, Gene Ontology (GO), InterPro, and KEGG PATHWAY. Comprehensive annotations for each gene are available at Supplementary Data 20.

### Identification of virus-like sequences

To identify putative VLSs, the following steps were performed: First, the *T. hydatigena* genome was searched against viral gene sequences from ViPR (https://www.viprbrc.org) by TBLASTN (E-value ≤ 1e^−3^). After removing redundant sequences, hit sequences with the longest predicted ORFs were translated into peptide sequences according to the blast results. Second, these candidate viral-like peptide sequences were aligned to the ‘nr’ database using BLASTP (E-value ≤ 1e^−4^). Only sequences of which the best hit was unambiguously annotated as a viral sequence were retained as putative ‘viral-like elements’. To evaluate the expression levels, the sequencing depth and coverage of virus elements were calculated using the protocol based on HISAT2 (v2.0.4)^51^ (default settings) and Samtools (v0.1.19) (default settings). The presence of a *rep* gene-like and a *cap* gene-like sequences in scaffold 2 of the *T. hydatigena* genome was validated by PCR amplification (forward primer: TCGAAACACAGAAACATGGAAGCC and reverse primer: CCGGAATTTCTTTGGTCTGGACAA; annealing temperature of 54 °C) (Supplementary Fig. 5).

### Alternative splicing analysis

After filtering the low-quality reads and adapter sequences, the clean RNA-seq reads of each sample were mapped to the *T. hydatigena* genome using HISAT2 (v 2.0.4) with default parameters. Alternative splicing in different samples was identified according to methods in the previous study^52^. Briefly, empirical transcripts were constructed using Cufflinks (v2.1.1)^53^ and alternative splicing events were identified using ASTALAVISTA (v4.0)^54^. The main types of alternative splicing events, including IR (intron retention), ES (exon skipping), AA (alternative acceptor site), and AD (alternative donor site) were annotated based on the definition that was previously described^55^.

In addition, RNA-seq data for different developmental stages of other flatworms were also used to identify alternative splicing events using the above-mentioned protocol. These included *T. saginata* (Bio-project IDs in GenBank: PRJNA260140 and PRJNA71493), *Clonorchis sinensis* (PRJNA386618 and PRJNA80037), *S. mansoni* (PRJEB15637, PRJEB2350, PRJNA236156, PRJNA361136, and PRJNA602007), *S. japonicum* (PRJNA515567 and PRJNA579703), and *T. solium* (PRJNA328007) (Supplementary Data 21). Weighted Gene Co-expression Network Analysis (WGCNA) for 15 tissues at different developmental stages was performed using the R package ‘WGCNA’^56^. The ‘mergedColors’ function in the package was used to merge profiles for modules with highly similar expressions. GO enrichment analysis of expanded gene families was performed using the EnrichPipeline^57^.

### Identification of metallopeptidase M08 family

In order to identify M08 peptidase in flatworms, the genomes of *T. asiatica*, *E. multilocularis, S. mansoni, Hymenolepis microstoma*, *S. japonicum*, *Schmidtea mediterranea*, and *C. sinensis* were searched by TBLASTN (E-value ≤ 1e^−5^ and identity ≥ 30%) using ‘invadolysin’ or ‘leishmanolysin’ amino acid sequences that were collected from ‘nr’ database (E-value ≤ 1e^−5^). Solar (v0.9.6)^27^ and GeneWise (v2.4.1)^47^ were used to identify the gene models based on the blast results in each species. The obtained genes were further confirmed by InterProScan for domain identification. Only candidates that have typical ‘invadolysin’ or ‘invadolysin-like’ domains (Pfam: PF01457) were used in this study. The gene structure of members in M08 peptidase family in the *T. hydatigena* genome was manually curated by the steps below: first, TBLASTN and GeneWise were used to predict the primary gene model of the gene using a homologous-based method; second, the exon–intron conjunction sites were verified by the results of RNA-seq data analysis from HISAT2 and Cufflinks; third, the entire gene structure was manually curated using the Integrative Genomics Viewer (IGV)^58^ based on the read alignments of RNA-seq data.

Amino acid sequences of M08 peptidase family in all species were aligned using Mafft (v7.427)^59^ (–auto). Sequences with extensive gaps (> 85%) or limited lengths (<200 aa) were excluded from the alignment. In the alignments, positions with large fractions of gaps (>20%) were trimmed using trimAl (1.2rev59)^60^. The best-fit model of evolution was selected by ProtTest (v 3.4.2)^61^ (WAG + Gamma + I). Phylogenetic relationships were inferred using maximum likelihood implemented in PhyML (version 3.0)^62^ with bootstrap replicates of 500.

### Identification of molecular targets for diagnosis and intervention

To identify GPCRs, the known GPCR protein sequences of helminths, *S. mansoni*, *S. mediterranea*, *Brugia malayi*, *Opisthorchis viverrini*, *E. multilocularis*, *T. asiatica,* and *T. saginata*^12,14,63^ were used as queries to search gene models of *T. hydatigena* using BLASTP (E-value ≤ 1e^−5^, identity ≥ 30%). The obtained blast hits were further searched against the ‘nr’ database by BLASTP (E-value ≤ 1e^−5^) to confirm their annotations. Genes that were homologs to other proteins in ‘nr’ annotations were excluded. Protein kinases and LGICs were identified using the same protocol.

In order to compare the homologous genes and identify specific genes between *T. hydatigena* and *T. solium* (PRJNA170813.WBPS14) or *E. granulosus* (PRJEB121.WBPS11), the genes in the *T. hydatigena* genome were compared with the genes of each species (*T. solium* and *E. granulosus)*: first, the gene models of *T. hydatigena* were searched against the gene models of *T. solium* (or *E. granulosus*) by BLASTP (E-value ≤ 1e^−5^, identity ≥ 50%); second, the genes without positive hits were further searched against the genome sequence of *T. solium* (or *E. granulosus*) by TBLASTN (E-value ≤ 1e^−5^, identity ≥ 50%) to further identify the homologous sequences. Only the genes with no positive hits in the two searches were considered a species-specific gene.

### Gene family and phylogenetic analysis

Gene families were constructed according to OrthoMCL^64^ with the parameter of ‘-inflation 1.5’ (OrthoMCL, RRID:SCR 007839). The phylogenetic relationship between *T. hydatigena* and other worms (*T. asiatica* (PRJNA299871), *T. saginata* (PRJNA71493), *T. solium* (PRJNA170813), *E. granulosus* (PRJEB121), *E. multilocularis* (PRJEB122), *H. microstoma* (PRJEB124), *S. mediterranea* (PRJNA12585), *S. mansoni* (PRJEA36577), *C. sinensis* (PRJDA72781), *O. viverrini* (PRJNA222628), *Fasciola hepatica* (PRJEB6687), *Gyrodactylus salaris* (PRJNA244375), and *Caenorhabditis elegans* (PRJNA13758)) was estimated based on concatenated single-copy orthologous genes (*n* = 253). In brief, the sequences of single-copy orthologous genes were firstly aligned by MUSCLE (v3.7)^65^ with default parameters, which were further concatenated to one supergene sequence for each species and formed a super distance matrix. Then the phylogenetic tree was inferred using maximum-likelihood (ML) algorithm in RAxML (v2.2)^66^ with the optimal amino acid substitution model selected by the PROTGAMMALGX parameter.

### Expansion and contraction of gene families

Divergence time was estimated using a Monte Carlo Markov Chain algorithm implemented in MCMCtree tool of the PAML package (v4.7)^67^. The divergence times from TimeTree (http://www.timetree.org/) were used to calibrate the divergence times of nodes on the phylogenetic tree: 25.0~30.0 million years ago (Ma) for *Taenia* and *Echinococcus*, 1.0~1.2 Ma for *T. saginata* and *T. asiatica*, and 570.0~1000.0 Ma for *C. elegans* and *S. mediterranea*. This time-calibrated phylogenetic tree was further used for the analysis of gene family expansion and contraction.

CAFÉ (v4.0)^68^ was used to analyze the expansion and contraction of orthologous gene families between ancestors and each of the 14 species (*T. hydatigena* and the above-mentioned 13 species in phylogenetic analysis) using a stochastic birth and death model with lambda potio. This model was further employed to infer changes of gene families along each lineage on the phylogenetic tree. A probabilistic graphical model was introduced to calculate the probability of transitions in gene family size from parent to child nodes. The corresponding *p*-values were calculated in each lineage based on the conditional likelihood. The abnormal gene families (families with gene numbers ≥200 in one species and ≤2 in all the other species) in individual species were excluded from the CAFÉ analysis. GO enrichment analysis of expanded gene families was performed using the EnrichPipeline^69^. *p*-values were corrected using FDR.

### Positive selection analysis

Genes under positive selection along the *T. hydatigena* lineage were identified by comparing with the tapeworms *T. hydatigena*, *T. asiatica*, *T. saginata*, and *T. solium*. Multiple sequence alignment of the single-copy genes identified from above four species was performed using MUSCLE. The signal of positive selection was detected by the branch-site model in codeml program implemented in PAML package (v4.7)^67^ (model = 2, NSsites = 2, kappa = 2.5, ncatG = 4).

### Statistics and reproducibility

Statistical analysis was performed by the two-sided Student’s *t*-test (confidence level = 0.95) or two-sided Wilcoxon rank sum test (confidence level = 0.95) using R. The *p*-values < 0.05 or FDR < 0.1 were considered to be significance. Reproducibility including biological independent sample sizes and replicates are stated in corresponding parts of Methods.

### Reporting summary

Further information on research design is available in the Nature Research Reporting Summary linked to this article.

## Supplementary information

Supplementary information.

Description of Supplementary Files.

Supplementary Data.

Reporting summary.

## Data Availability

The whole Genome Shotgun project of *T. hydatigena* and all raw sequencing data for the version 1.2 have been deposited at NCBI/BioProject: PRJNA671790. The new version of genome assembly (version 2.1) and raw Hi-C sequencing data have been deposited at NCBI/BioProject: PRJNA734747. All files for CDS, protein sequences, alternative splicing events and annotations for the protein-coding genes of the *T. hydatigena* genome (version 1.2 and version 2.1) and the source data for each figure are available at Open Science Foundation (https://osf.io/hb7w8/?view_only=f77fd04ccd904e7e8c0f0a02370b282e). Any remaining data are available from the corresponding author upon reasonable request.
