# Supplementary material for: The genome of the thin-necked bladder worm Taenia hydatigena reveals evolutionary strategies for helminth survival
Source: Commun Biol. 2021 Aug 24;4:1004. doi: 10.1038/s42003-021-02536-w (PMC8384839; doi:10.1038/s42003-021-02536-w)
Supplement: Supplementary file 1 — Supplementary information. [file 42003_2021_2536_MOESM1_ESM.pdf]

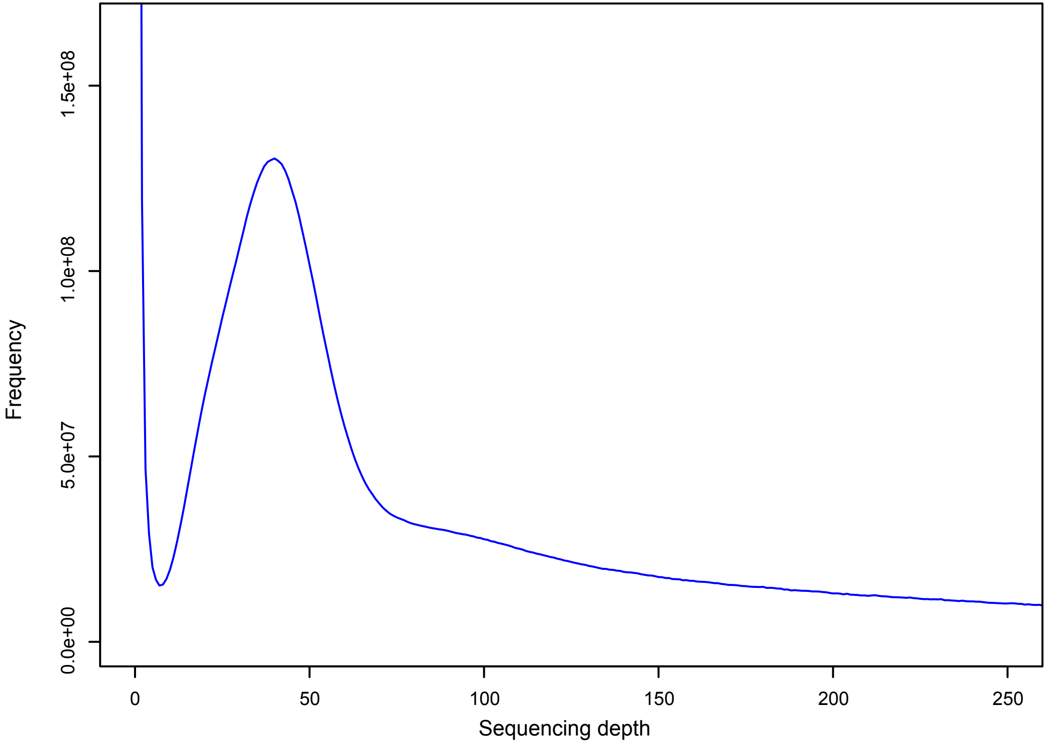

Supplementary Figure 1. Frequency distribution of 17-mers of the sequenced pair-end reads. The genome size of *T. hydatigena* was estimated with the formula used in the reference[27].

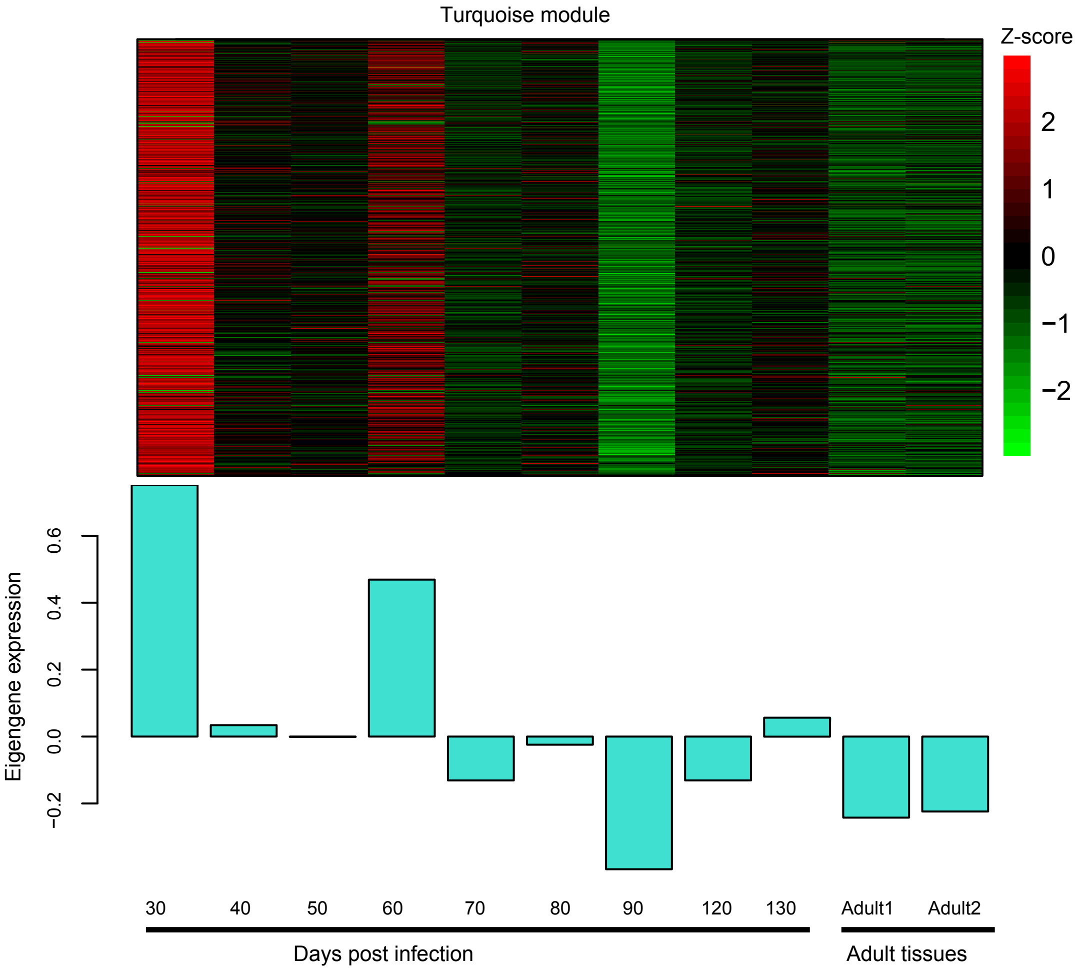

Supplementary Figure 2. Comparison of eigengene expressions of the turquoise module. The result for different developmental stages (larvae collected at days post infection and adults) are shown based on the results from WGCNA.

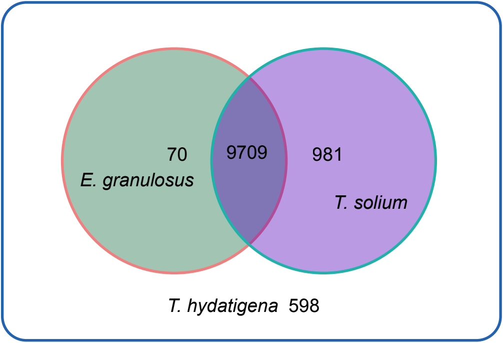

Supplemenatry Figure 3. Comparison of gene sets for the relevant tapeworms that share the intermediate hosts with *T. hydatigena*. The comparisions were based on BLAST searches (See Methods).

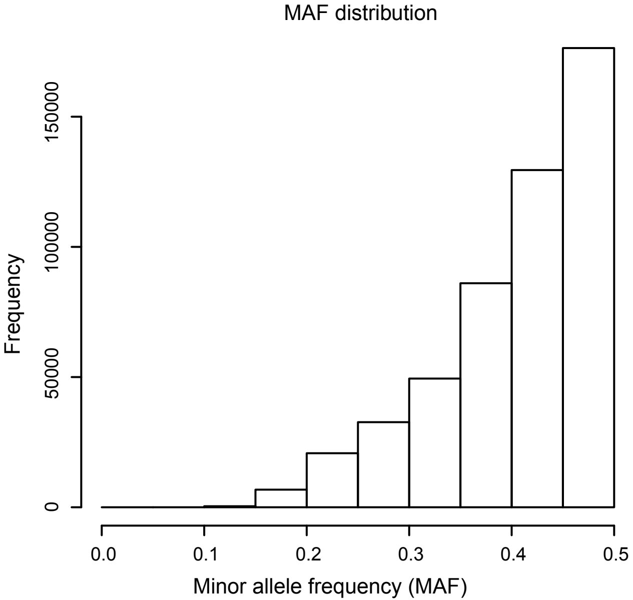

Supplementary Figure 4. Distribution of minor allele frequency of the *T. hydatigena* isolate. The distribution of MAF peaks at 0.5 for the genome, which indicates it is diploid.

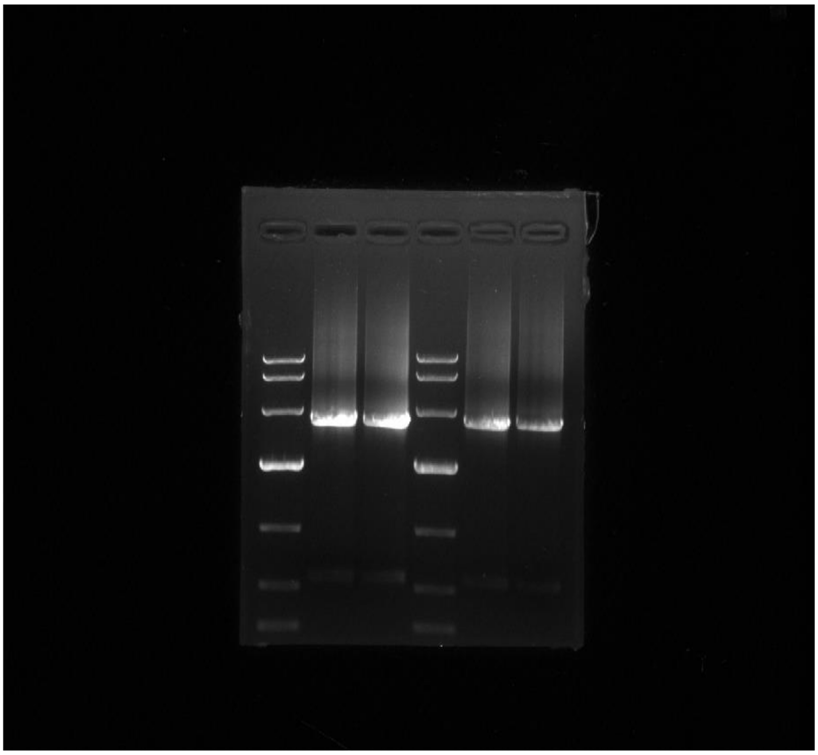

Supplementary Figure 5. Uncropped gel image for Figure 2c.

Supplementary Note 1. The Hi-C data and the genome assembly v2.1.

Hi-C library sequencing and scaffolding

The Hi-C sequencing libraries were prepared according to the standard protocol described previously1 and sequenced on Illumina NovaSeq 6000. After sequencing, the raw sequencing data was filtered for adapters, reads with N bases of more than 10%, and reads with low-quality bases (≤ 5) of more than 50%. Totally, 33.11 Gb high-quality clean data (~98 × coverage depth) were produced. Based on the linkage information of proximity ligation from the Hi-C data, the assembled scaffolds (version 1.2) were mounted to the chromosome level using 3D-DNA software[2] (longest_=800, shortest_=150, NonInformativeRatio = 0, minREs = 50, MaxLinkDensity = 3, r = 2, i = 15000, editor-repeat-coverage = 4) to obtain a new version of genome assembly (version 2.1).

The protein-coding genes and non-coding genes were lifted over from the version 1.2 to version 2.1 by coordinating the linkage information between the two genome assemblies in the AGP file, which was generated during Hi-C data assembling. Repetitive elements within the genome assembly v2.1 were identified using the RepeatMasker library constructed from the genome assembly v1.2.

Statistics of the genome assembly v2.1

The genome assembly has been deposited at NCBI/BioProject: PRJNA734747. The statistic results of the *T. hydatigena* genome assembly (version 2.1):

	**Contig length**	**Scaffold length**	**Contig number**	**Scaffold number**
Total	307,042,196	308,614,463	4,215	1,921
Max	8,067,506	36,935,678	-	-
N50	170,760	18,789,986	327	7
N60	120,173	16,259,214	543	8
N70	85,000	579,000	852	18
N80	62,947	144,000	1,271	165
N90	40,655	67,000	1,867	496

Supplementary Note 2. The repetitive elements and genome features of the genome assembly version 2.1.

The repetitive elements in the *T. hydatigena* genome (v2.1).

Type	Length (bp)	% of the genome
DNA	9,645,706	3.13
LINE	2,897,282	0.94
SINE	6,900,654	2.24
LTR	45,156,309	14.634
Satellite	1,417,638	0.46
Simple_repeat	4,091,663	1.33
Unknown	144,757,939	46.91
Total	196,564,717	63.69

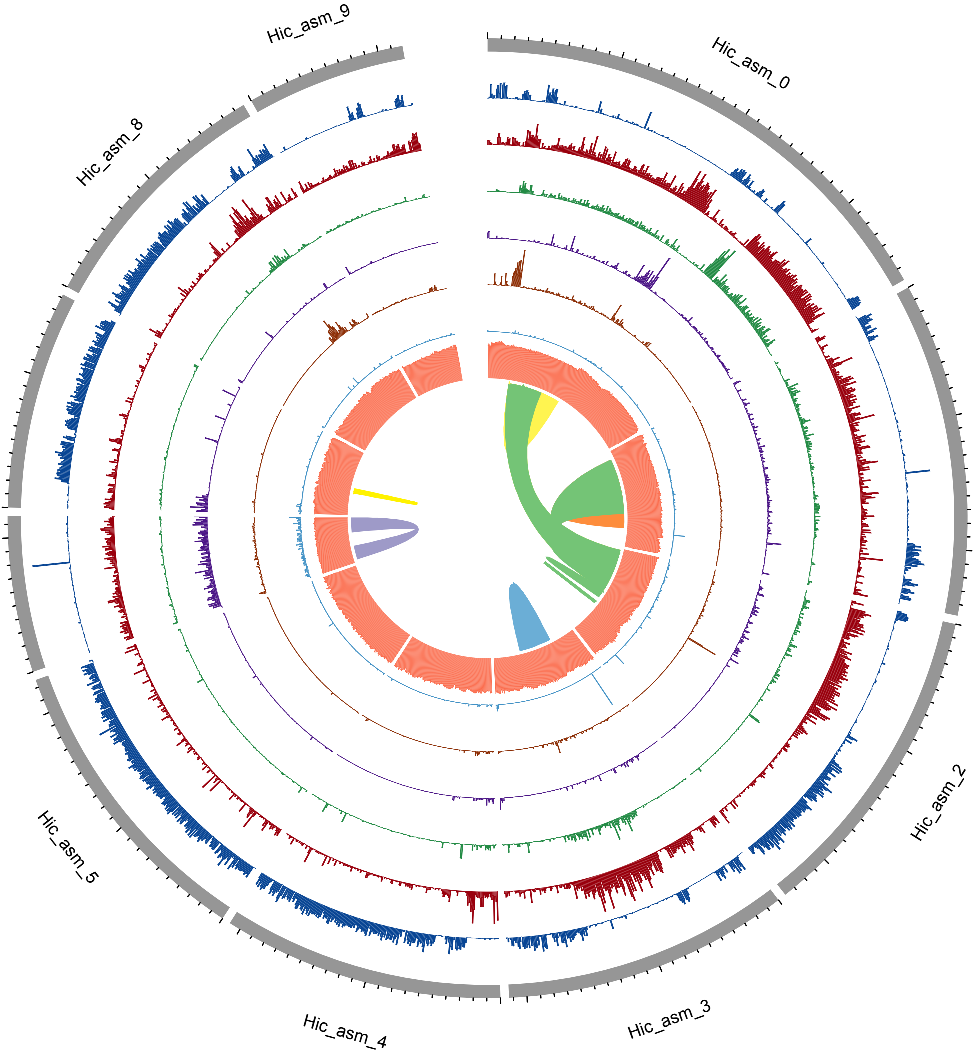

Circular overview of the *T. hydatigena* genome (v2.1). Circular overview plot shows basic features across the *T. hydatigena* genome assembly (v2.1). This genome assembly was assembled using Hi-C technique. From outermost to innermost ring: density of gene models, density of long terminal repeat retrotransposons, density of DNA transposons, density of short interspersed nuclear elements, density of long interspersed nuclear elements, density of non-coding RNA, % of GC, and segmental duplications (≥ 5 genes in a block). All tracks except scaffold alignments show binned data with a window size of 0.1 Mb. The top 10 scaffolds in length are shown.

Supplementary References

1. Belton JM, McCord RP, Gibcus JH, Naumova N, Zhan Y, Dekker J. Hi-C: a comprehensive technique to capture the conformation of genomes. *Methods* 2012, **58**(3)**:** 268-276.

2. Dudchenko O, Batra SS, Omer AD, Nyquist SK, Hoeger M, Durand NC*, et al.* De novo assembly of the Aedes aegypti genome using Hi-C yields chromosome-length scaffolds. *Science* 2017, **356**(6333)**:** 92-95.
