# Supplementary material for: The genome of the thin-necked bladder worm Taenia hydatigena reveals evolutionary strategies for helminth survival
Source: Commun Biol. 2021 Aug 24;4:1004. doi: 10.1038/s42003-021-02536-w (PMC8384839; doi:10.1038/s42003-021-02536-w)
Supplement: Supplementary file 2 — Description of Supplementary Files. [file 42003_2021_2536_MOESM2_ESM.pdf]

Description of Additional Supplementary Files

File name: Supplementary Data

Description:

Supplementary Data 1: The host records for T. hydatigena in NHM host-parasite database.

Supplementary Data 2: Statistics of the T. hydatigena genome assembly.

Supplementary Data 3: Summary of coding gene annotations for the T. hydatigena genome.

Supplementary Data 4: Summary of non-coding genes in the T. hydatigena genome.

Supplementary Data 5: Summary of repetitive elements in the tapeworm genomes.

Supplementary Data 6: The repetitive elements labeled with 'Class=Unknown' in the T. hydatigena genome.

Supplementary Data 7: Virus-like sequences identified in the genome.

Supplementary Data 8: FPKM values of the virus-like sequences in the genome.

Supplementary Data 9: The gene IDs of the 'invadolysin' coding genes in tapeworms.

Supplementary Data 10: The genes of each module in WGCNA analysis.

Supplementary Data 11: Enriched Go terms for the genes in the module 'turquoise'.

Supplementary Data 12: Expanded gene families in the T. hydatigena genome.

Supplementary Data 13: Positively selected genes identified in the T. hydatigena genome.

Supplementary Data 14: The species-specific protein-coding genes (versus T. solium) in the T. hydatigena genome.

Supplementary Data 15: The species-specific protein-coding genes (versus E. granulosus) in the T. hydatigena genome.

Supplementary Data 16: The species-specific protein-coding genes (versus both T. solium and E. granulosus) in the T. hydatigena genome.

Supplementary Data 17: G-protein-coupled receptors (GPCRs) identified in the genome.

Supplementary Data 18: Protein kinases identified in the genome.

Supplementary Data 19: Ligand-gated ion channels (LGICs) identified in the genome.

Supplementary Data 20: Comprehensive annotations of the protein-coding genes.

Supplementary Data 21: Transcriptome data used in the analysis of M08 peptidase gene family.
